# A MALDI-ToF mass spectrometry database for identification and classification of highly pathogenic bacteria

**DOI:** 10.1038/s41597-025-04504-z

**Published:** 2025-01-31

**Authors:** Peter Lasch, Wolfgang Beyer, Alejandra Bosch, Rainer Borriss, Michal Drevinek, Susann Dupke, Monika Ehling-Schulz, Xuewen Gao, Roland Grunow, Daniela Jacob, Silke R. Klee, Armand Paauw, Jörg Rau, Andy Schneider, Holger C. Scholz, Maren Stämmler, Le Thi Thanh Tam, Herbert Tomaso, Guido Werner, Joerg Doellinger

**Affiliations:** 1 Robert Koch Institute, ZBS 6 - Proteomics and Spectroscopy, Seestraße 10, Berlin, D-13353 Germany; 2 Advisory Panel of the Medical Academy of the German Armed Forces, Bundeswehr Institute of Microbiology, Munich, Germany; 3 CINDEFI-UNLP-CONICET, CCT La Plata, Facultad de Ciencias Exactas, Universidad Nacional de La Plata, La Plata, Buenos Aires, Argentina; 4 Institute of Marine Biotechnology e.V. (IMaB), Greifswald, Germany; 5 National Institute for Nuclear, Chemical and Biological Protection, Milin, Czech Republic; 6 Robert Koch Institute, ZBS 2 - Highly Pathogenic Microorganisms, Berlin, Germany; 7 Functional Microbiology, Institute of Microbiology, University of Veterinary Medicine, Vienna, Austria; 8 College of Plant Protection, Nanjing Agricultural University, Key Laboratory of Integrated Management of Crop Diseases and Pests, Nanjing, People’s Republic of China; 9 Netherlands Organization for Applied Scientific Research TNO, Department of CBRN Protection, Rijswijk, The Netherlands; 10 Chemisches und Veterinäruntersuchungsamt Stuttgart (CVUAS), Fellbach, Germany; 11 Division of Plant Pathology and Phyto-Immunology, Plant Protection Research Institute, Hanoi, Vietnam; 12 Friedrich-Loeffler-Institut (FLI), Federal Research Institute for Animal Health, Jena, Germany; 13 Robert Koch Institute, Nosocomial Pathogens and Antibiotic Resistances (FG13) and National Reference Centre for Staphylococci and Enterococci, Wernigerode, Germany

**Keywords:** Bacterial techniques and applications, Classification and taxonomy, Clinical microbiology

## Abstract

Today, MALDI-ToF MS is an established technique to characterize and identify pathogenic bacteria. The technique is increasingly applied by clinical microbiological laboratories that use commercially available complete solutions, including spectra databases covering clinically relevant bacteria. Such databases are validated for clinical, or research applications, but are often less comprehensive concerning highly pathogenic bacteria (HPB). To improve MALDI-ToF MS diagnostics of HPB we initiated a program to develop protocols for reliable and MALDI-compatible microbial inactivation and to acquire mass spectra thereof many years ago. As a result of this project, databases covering HPB, closely related bacteria, and bacteria of clinical relevance have been made publicly available on platforms such as ZENODO. This publication in detail describes the most recent version of this database. The dataset contains a total of 11,055 spectra from altogether 1,601 microbial strains and 264 species and is primarily intended to improve the diagnosis of HPB. We hope that our MALDI-ToF MS data may also be a valuable resource for developing machine learning-based bacterial identification and classification methods.

## Background & Summary

Matrix-assisted laser desorption/ionization time-of-flight mass spectrometry (MALDI-ToF MS) is a powerful analytical technique used for rapidly identifying and characterizing biomolecules, particularly proteins and peptides. In the realm of clinical microbiology, MALDI-ToF MS plays a crucial role today in the swift and accurate identification of pathogenic bacteria^1,2^.

**Fig. 1:**
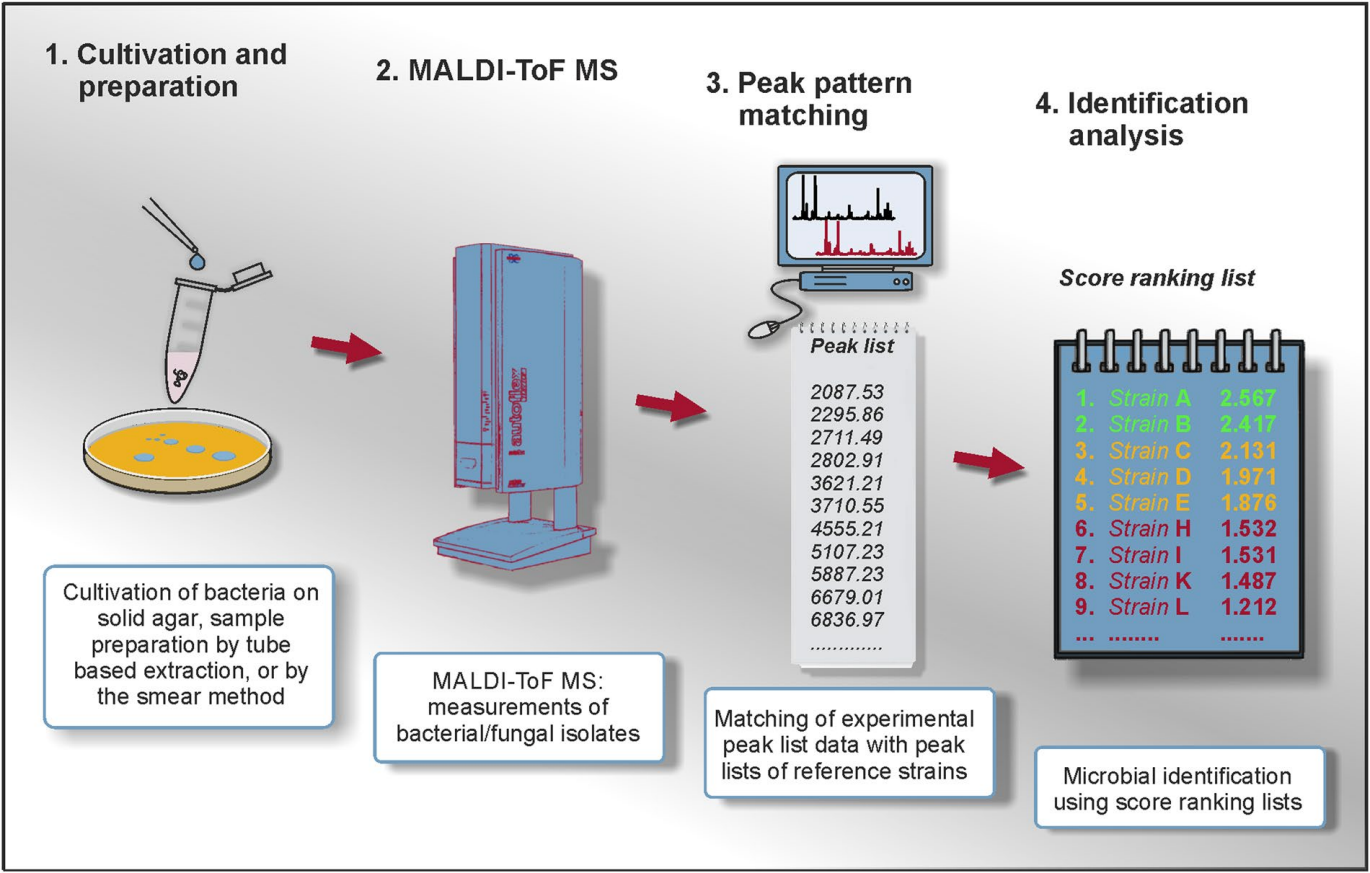
General workflow of MALDI-ToF mass spectrometry-based identification analysis.

The workflow for MALDI-ToF MS-based identification of microorganisms involves the growth of bacterial colonies, usually on solid media as an initial step (for an illustration of the principal MALDI-ToF MS workflow see Fig. 1). In the simplest case, material from a single colony is obtained and directly smeared onto a target before covering it with matrix solution (smear, or intact cell technique). After drying the bacterial material, mass spectra can be acquired, which provide information about the mass-to-charge ratio (m/z), mostly of small basic ribosomal subunit proteins consistently present in high amounts in microbial cells^3^. The mass spectra obtained are then compared to a validated reference database of MALDI-ToF mass spectra from microorganisms with a known taxonomic status. Through pattern matching, the specific unique mass fingerprints facilitate the identification of the microorganism, with the degree of match indicating the taxonomic level of identification^4^. In the last decade, this rapid, accurate, and high throughput workflow has become standard in clinical microbiology for the timely and precise identification of pathogenic bacteria and fungi^5–7^.

In the context of this workflow, the importance of mass spectral databases cannot be overstated. These databases contain reference spectra from known microorganisms for pattern matching and are fundamental for comparison and identification. They play a crucial role in enabling rapid and accurate identification of microbial isolates, for example, in clinical microbiology where timely decision-making for patient treatment is vital. Database content should be dynamic and must be updated on a regular basis by new entries to cover emerging microorganisms and to take the latest taxonomic classification system into account^8^.

In addition to its broad application in routine clinical microbiology, where accurate identification, reduced costs, automation options and high sample throughput are essential, the MS-based workflow for bacteria has also established itself in other application areas. These include environmental microbiology^9^, food quality control^10^, veterinary medicine^11^, application by academic users, and biosecurity applications such as the diagnostics of highly pathogenic bacteria mainly grouped in biosafety level 3 (BSL-3)^12,13^.

Many of the authors of this study were involved in the latter activity. With the establishment of the Centre for Biological Threats and Special Pathogens (ZBS) at the Robert Koch Institute (RKI) in 2002, a comprehensive program for the characterization of BSL-3 pathogens using MALDI-ToF MS has been initiated. The main goal of this program was to establish a mass spectrometry-based method for the rapid, reliable, and accurate identification of bioterror-relevant highly pathogenic bacteria (HPB) that complements established bacterial identification methods such as species-specific PCRs, 16S rRNA sequencing, or whole-genome sequencing (WGS). As part of this program, a protocol for secure and MS-compatible inactivation of HPB has been developed and implemented, which assures complete inactivation of bacterial samples that may contain very high concentrations of bacterial endospores (e.g. spores of *Bacillus anthracis*)^14,15^. After successfully completing this first project task, we started with compiling spectral databases from HPB and related strains and species in 2007. Within the frame of these efforts, the previously developed inactivation protocol was applied to BSL-3 pathogens such as *Bacillus anthracis*^16^, *Yersinia pestis*^17^, *Francisella tularensis*, *Burkholderia mallei/pseudomallei*^18^ as well as to a large number of their close and more distant relatives. As with the application of MALDI-ToF MS in clinical microbiology, the success of the MS method for HPB identification is highly dependent on the availability of mass spectra, ideally acquired under standardized conditions and covering relevant species of microbial genera containing BSL-3 pathogens.

In the following years, spectral databases established at RKI were continuously expanded. For this purpose, bacteria from RKI strain collections and from partners in Argentina^19,20^, China^21^, Vietnam^22^, and from European collaborators were characterized and added^23–25^. In this context, a European quality assurance exercise should be emphasized, in which a total of eleven partner institutions from nine European countries systematically investigated the possibilities of diagnosing HPB with MALDI-ToF MS under blinded conditions^13^. The results of this collaborative exercise demonstrated that the success of the methodology is mainly based on the quality of the underlying databases.

A significant result of all these activities was the decision to make the RKI’s MALDI-ToF MS databases publicly available. This decision was further strengthened by publications reporting MALDI misdiagnoses^26^ resulting from missing, or inadequate entries of HPB in commercial databases. Such wrong identification results had not only a direct impact on the treatment of patients, but also significantly disrupted routine procedures in the affected diagnostic facilities^27–30^. In addition, the RKI’s human reference laboratory for *Bacillus anthracis* was contacted several times by users of the Security-related (SR) library extension, an additional component of Bruker’s commercial MALDI Biotyper (MBT) solution, who observed false positive identifications of *B. cereus* or *B. thuringiensis* isolates as *B. anthracis*. Although these misidentifications did not put patients or clinical staff at risk, the use of improved HPB databases would obviously have saved time, resources, and effort, since anthrax is a very harmful and notifiable disease.

Based on these considerations, four versions of the MALDI-ToF MS database have been published since 2016 on ZENODO, a general-purpose open repository developed under the European OpenAIRE program and operated by CERN. Spectra from other published MALDI-ToF MS studies were included in addition to the above-mentioned HPB spectra generated from the RKI and its collaborators^19,21,22,31–34^. The publication of the RKI’s databases was met with a broad response; by December 2024 the download statistics of the ZENODO data repository showed a total of more than 6500 downloads across all database versions. In addition, spectra from the RKI database are also available from other MALDI repositories, such as the MALDI-UP Spectra Catalog^35^ maintained by the CVUAS (Chemisches und Veterinäruntersuchungsamt Stuttgart, Germany), or can be used for online identification analysis through MicrobeNet^36^, a web service hosted by the Centers for Disease Control and Prevention (CDC) and curated by CDC experts (https://microbenet.cdc.gov/). MicrobeNet was developed as a free service to provide detailed information on rare and unusual pathogens, including bacteria and fungi.

As the data is freely accessible and can be used without technical restrictions, the authors of this study have only very limited information about whether and to what extent the frequent downloads ultimately led to their use in everyday clinical practice. We found one example of the use of our database in a clinical setting in Suniga *et al*., 2023^37^. In addition to this primary purpose, spectra from the RKI database have also been used for academic research, most notably to train machine learning models for MALDI-ToF MS-based identification analysis, or in extensive validation studies for the use of MALDI-ToF MS in official food control laboratories^20,38–40^.

In this article, we report 11,055 MALDI-ToF mass spectra comprising version 4.2 of the RKI database identifying and classifying HPB^41^. The spectra were acquired from 73 bacterial genera and cover a total of 264 species and 1601 strains. The aim of this publication is to introduce our database to a wider audience and to provide a comprehensive description of the dataset. It is our hope that this will encourage the future use of freely accessible MALDI-ToF MS databases in both, diagnostics beyond routine and academic research.

## Methods

The ZENODO spectral database^41^ contains a vast number of microbial mass spectra that were collected by the RKI and its partner institutions over a period of almost 20 years. Therefore, it is not possible to describe all details of cultivation, sample preparation, or data acquisition for each spectrum acquired. However, in the ZENODO MALDI-ToF spectrum repository we have provided a PDF file that contains metadata entries for each individual spectrum and details taxonomic descriptors as well as selected sample preparation and data acquisition parameters^41^. In the following, we will thus focus on describing the typical workflow used by the RKI group for the acquisition of MALDI-ToF mass spectra from bacterial isolates.

### Cultivation

Samples were cultivated on solid agar media appropriate for the respective species. Typically, cells were grown as pure cultures for two passages under aerobic conditions and then harvested by adding the equivalent of three full 1 µL plastic loops (approximately 4 mg) to 20 µL of sterile water; for details, see publications on the trifluoroacetic acid (TFA) inactivation protocol^14,15^. Further cultivation details (temperature, duration, medium composition, etc.) along with other metadata can be retrieved for each MALDI-ToF mass spectrum record from the ZENODO website^41^.

### Sample preparation

Microbial sample preparation at RKI was performed using the TFA protocol, ensuring complete and MALDI-ToF MS compatible inactivation of the bacteria investigated, including bacterial endospores^14^. Briefly, a volume of 80 µL of pure TFA was added to the above-mentioned microbial suspension. After 30 min of incubation, the solutions were diluted tenfold with HPLC grade water. The microbial sample solutions were then mixed with a highly concentrated α-cyano-4-hydroxycinnamic acid (HCCA) solution (12 mL/mL) prepared by dissolving HCCA in TA2, a 2:1 (v/v) mixture of 100% acetonitrile and 0.3% TFA. Two microliters of these mixtures were spotted onto steel sample targets prior to the measurements.

The ethanol-formic acid protocol developed by Bruker Daltonics, which is a standard protocol in MS-based microbial diagnostics^42^, was often used for sample preparation by the RKI’s collaborators. Again, specific details of the sample preparation protocols employed are detailed in the metadata document available from the ZENODO website^41^.

### MALDI-ToF MS measurements

At RKI, mass spectral profiles of microbial preparations were obtained using an *autofleX I* mass spectrometer from Bruker Daltonics. The instrument is equipped with a nitrogen UV laser operating at 337 nm, which was slightly defocused for better signal quality. The pulse ion extraction time was set to values of 200 ns, or 300 ns for measurements conducted after 2014. All measurements were performed in linear positive mode. The acceleration voltage was set to 20.00 (ion source 1) or 18.45 (ion source 2) kV, while the lens voltage was 6.70 kV. Mass spectra were stored in the m/z range between 2000 and 20,000. Linear or quadratic calibration was carried out by using Bruker’s Protein Calibration Standard I (measurements prior to 2011), or the *Escherichia coli* DSM 3871 reference strain for measurements after that date. For external calibration with *E. coli*, peaks of the following bacterial proteins were utilized: doubly charged 50S ribosomal protein L29 (RL29 at m/z position 3637.8; [M + 2H]^2+^), RL36 (m/z 4365.3, [M + H]^+^), RS22 (m/z 5096.8, [M + H]^+^), RL34 (m/z 5381.4, [M + H]^+^), RL33meth (m/z 6255.6, [M + H]^+^), RL32 (m/z 6316.2, [M + H]^+^), RL30 (m/z 6411.6, M + H]^+^), RL35 (m/z 7158.8, [M + H]^+^), RL29 (m/z 7274.5, [M + H]^+^), RL31 (m/z 7872.1, [M + H]^+^), RS21 (m/z 8369.8, [M + H]^+^), DNA-binding protein HU-beta (m/z 9226.6, [M + H]^+^), and RS20 (m/z 9536.3, [M + H]^+^)^33^. Calibration standards were always placed on 384 MTP reusable steel MALDI target plates immediately adjacent to the sample spots. All mass spectra were acquired in full manual mode by co-adding signals from at least 600 (before 2015), typically of 1000 individual laser shots (2015 and later).

## Data Records

The raw mass spectrometry data, the corresponding metadata information and peak list data required for identification analysis is available via the ZENODO data repository under the name “*Version 4.2 (20230306) of the MALDI-ToF Mass Spectrometry Database for Identification and Classification of Highly Pathogenic Microorganisms from the Robert Koch Institute (RKI)*”^41^ (10.5281/zenodo.7702374). Peak list data are available in two different formats, the *.btmsp format required by Bruker’s commercial MALDI Biotyper software solution and the *.pkf format suitable for identification analysis by MicrobeMS, a free software solution developed at RKI^43^.

### Database content

**Fig. 2:**
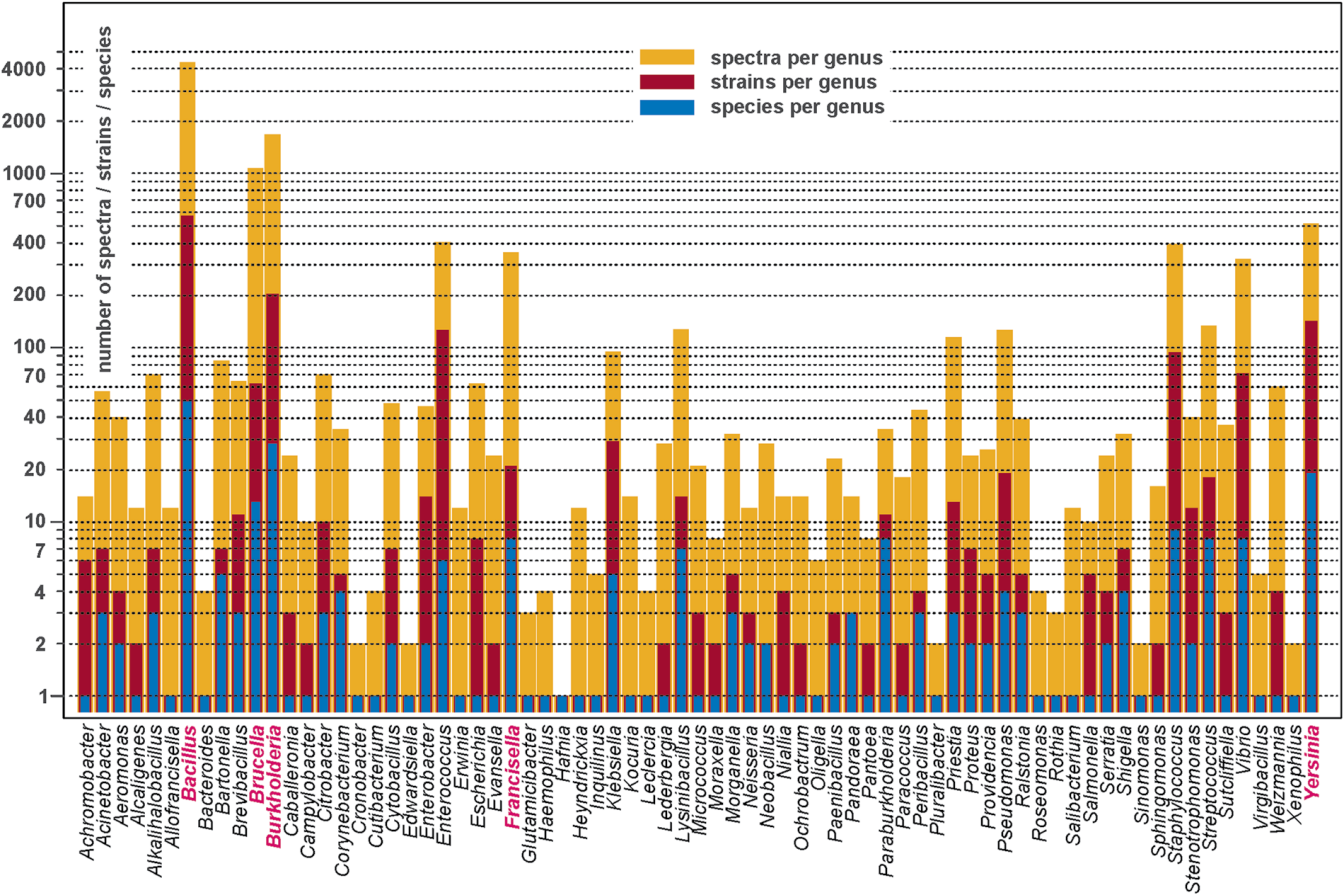
Bar chart giving an overview on the microbial genera represented in the ZENODO MALDI-ToF mass spectrometry database. The height of the bars illustrates the number of MALDI-ToF mass spectra per genus (dark yellow bars), the number of strains of the given genus (ruby red bars) as well as the number of microbial species per genus (blue bars). Note the logarithmic scaling of the y-axis. Further details on database composition are available from the ZENODO data repository, see MS Excel file *“Taxonomy information - RKI MALDI-ToF MS database of HPB at ZENODO v.4.xlsx*”^41^.

Version 4.2 of the RKI database contains 11,055 MALDI-ToF mass spectra in total which were collected from 73 microbial genera, 264 species and 1601 strains. Figure 2 shows a log-scaled bar chart giving an overview of the contents of the latest database version. While the height of the dark yellow bars illustrates the number of MALDI-ToF mass spectra per genus, the ruby red and blue bars show the number of strains and of the microbial species per genus, respectively. The genera with the highest number of database entries, i.e. *Bacillus, Brucella, Burkholderia, Francisella, and Yersinia*, contain one or more HPB species. An overview of the database composition is provided in sheet #1 of the Excel table entitled “*Taxonomy information - RKI MALDI-ToF MS database of HPB at ZENODO v.4.xlsx”* available from the data repository at ZENODO^41^. In this file, not only all spectra are listed, it details also the number of spectra per genus/species/strain, the number of strains per genus/species, and the number of species of each bacterial genus.

**Fig. 3:**
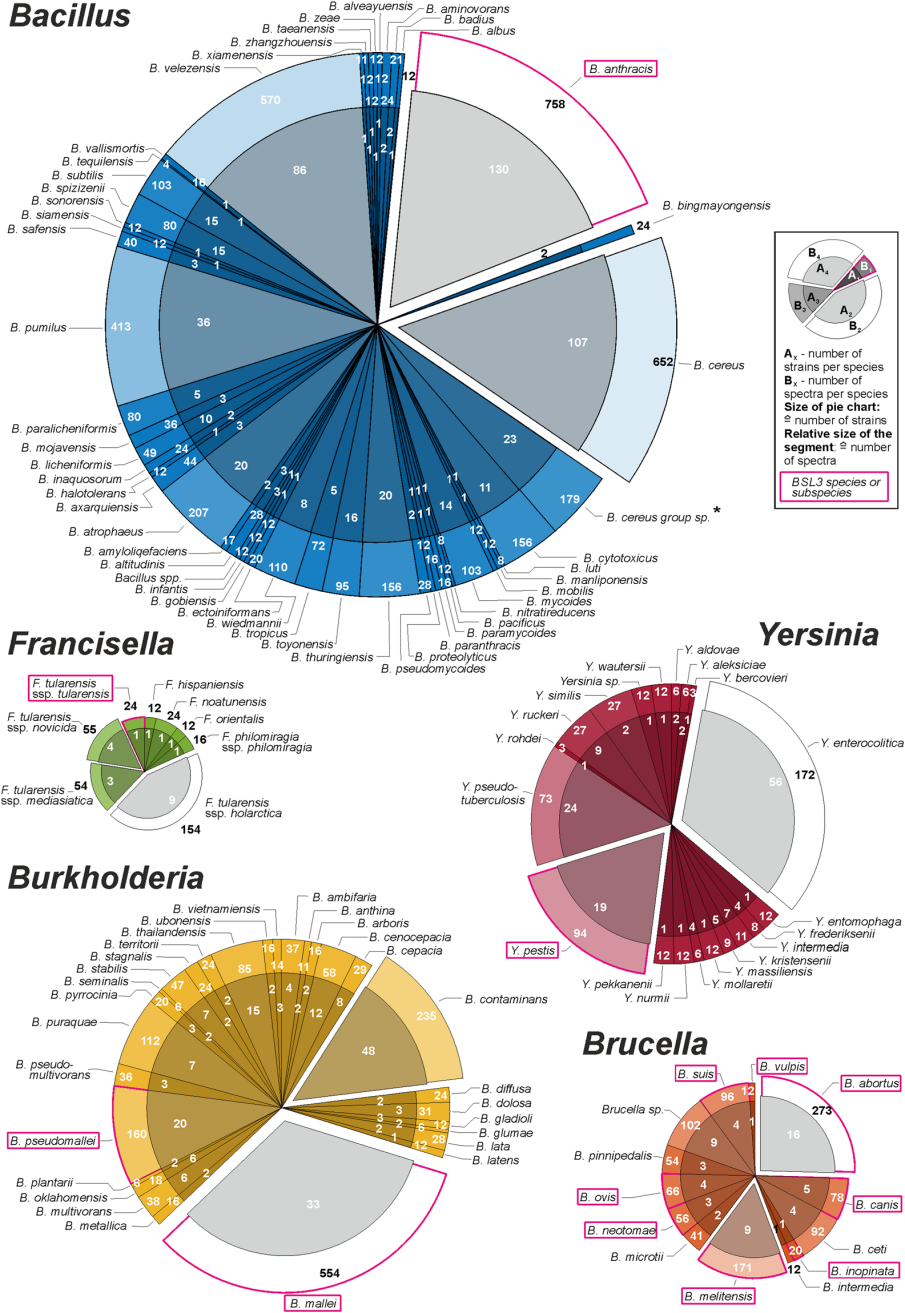
Pie charts illustrating database content by strains and species of selected genera containing highly pathogenic bacterial species: *Bacillus*, *Brucella*, *Burkholderia*, *Francisella*, and *Yersinia*. The size (area) of each pie chart is proportional to the number of strains of the given genus represented in the data base. Furthermore, each pie chart contains segments that provide further information. The size and color intensity of the individual segments are proportional, or inversely proportional, respectively, to the number of spectra recorded from the given species. The chart segments further contain information regarding the number of strains per species (numbers in the inner circles) and the number of spectra per species (outer circle). Names of highly pathogenic (BSL-3) microbial species or subspecies are plotted in pink framed text boxes. *Bacillus cereus* group sp.*: Members of the *B. cereus* group for which no species assignment is available.

The contents of the database for the most relevant and best-represented genera *Bacillus*, *Brucella*, *Burkholderia*, *Francisella* and *Yersinia* are shown in Fig. 3. In this figure, the size (area) of each genus-specific pie chart is proportional to the number of strains of that genus represented in the database. Furthermore, the size of the segments and their color intensities illustrate the number of spectra recorded from the given species. While the numbers of the inner parts indicate the number of strains of the given species, the numbers in the outer parts of the pie charts denote the respective number of MALDI spectra. Pink framed text fields indicate HPB.

### Raw spectral data

All raw spectral data, i.e. mass spectra, are available in the native proprietary Bruker Daltonics file format (zip archive of a size of approximately 1 GB). In this format, each individual spectrum of a microbial sample constitutes a rather complex directory structure containing at least seven files, either in binary format with time-of-flight data (‘fid’) or in simple text format (‘acqu’, ‘acqus’, ‘sptype’). Text files contain metadata, measurement parameters, calibration information, etc. Metadata, such as cultivation parameters, operator names, taxonomy data, etc., were added to the acqu and acqus text files. This latter data can be read using appropriate software (e.g. MicrobeMS) upon import of original MALDI data (see the MicrobeMS website^43^ for details). The free MicrobeMS software can be also used for converting Bruker spectrum data from the proprietary into open text formats.

After unpacking the zip archive, the directory structure of the database largely corresponds to the structure of the microbial taxonomy: Mass spectra of a given genus are located in a folder named after the genus, which then contains subfolders named after species, subspecies, and strains, respectively. Next, all MALDI-ToF technical replicate spectra of a biological replicate can be found in subfolders named like “*Messung x*” whereas the numerical value x denotes the index of the biological replicate.

### Metadata

Aside from the metadata stored in the original spectra files, the data collection deposited at ZENODO contains a metadata pdf document (230306-ZENODO-Metadata.pdf). In this text document, the following metadata are provided for each MALDI-ToF mass spectrum: taxonomy information, measurement date and time, NCBI taxonomy ID, growth time, growth temperature, growth medium and other growth conditions, sample amount, sample preparation method, calibration and extra information, measurement method, license and customer information, the path to MS files, Bruker and MicrobeMS ID. The pdf document comprises a total of 1686 pages!

The Excel file *Taxonomy information - RKI MALDI-ToF MS database of HPB at ZENODO v.4.xlsx* available from the ZENODO data repository^41^ contains additional taxonomic information such as (i) a detailed list of bacterial MALDI-ToF mass spectra (sheet #1), (ii) overviews on the number of spectra per strain, species, or bacterial genus (sheet #2), (iii) numbers of strains per species, or genus (sheet #3), and (iv) in sheet #4 the number of species per microbial genus.

### Peak list data

Peak lists are an important part of the data publication at ZENODO. Such lists are derived from original MALDI-ToF mass spectra and are available in Bruker Daltonics’ MBT (*btmsp*) and in the MicrobeMS data format (*pkf*).

Peak list files in the *btmsp* format are compressed (zipped) file containers containing XML-formatted text files representing MSPs required for identification analysis by Bruker’s proprietary MBT solution. The abbreviation MSP has two meanings in the literature: main spectrum or main spectral projection. MSPs can be considered database spectra that contain metadata and peak statistics derived from replicate spectra used to generate the MSP. Probably due to the size of the database, we were unable to import the single *btmsp* file initially created from the complete MSP selection. Therefore, the MBT peak list file of the ZENODO V.4.1 and V.4.2 database had to be split into several *btmsp* files. To use the complete database, it is thus necessary to download and import all of the provided *btmsp* files. At ZENODO, each *btmsp* file is named according to the following scheme: the first parts of the filename indicate the creation date, followed by the name of the genus and the number of MSPs contained. As an illustration, the file “*2023-May-23-Vibrio-RKI-Database-71.btmsp*” was created on May 23, 2023, and comprises 71 MSPs from the genus *Vibrio*. In cases where *btmsp* files contain MSPs from multiple genera, only the initial letters of the genera are indicated. To give a second example, the file “*2023-May-23-M-X-RKI-Database-252.btmsp*” comprises 252 MSPs derived from mass spectra of the genera *Micrococcus*, *Moraxella*,…, up to *Xenophilus*.

The peak list files “*230306_ZENODO_30Peaks_0.75.pkf”* and “*230306_ZENODO_45Peaks_0.75.pkf”* contain peak lists that constitute MicrobeMS database spectra in the m/z range 2000–13,000. The first *pkf* file has been calculated by setting the value “number of peaks” of the peak lists to 30. In the second file this number has been increased to 45. Each peak list entry contains, in addition to spectrum metadata, average m/z peak positions, peak intensity values and corresponding peak frequency information derived from sets of strain-specific mass spectra. The total number of peak lists comprising each *pkf* file equals 1601, corresponding to the total number of microbial strains in the ZENODO v.4.2 database. Peak list files in the *pkf* format were generated by the MicrobeMS software package and are required for MicrobeMS-based identification analysis. See the MicrobeMS Wiki for details on how to perform identification analysis using a *pkf* peak list file^43^.

## Technical Validation

### Data quality

A major advantage of MALDI-ToF MS measurements performed by experienced operators over automated measurement routines is the higher spectral quality that can be achieved. For example, the manual measurement mode allows the trained analyst to adjust the laser energy to the specific properties of the sample or to select sample regions with an optimal matrix/analyte ratio. In addition, although manual measurements can be time-consuming, they have the inherent advantage that subsequent evaluation and selection of spectra is generally not required, since the quality of the recorded spectra is directly assessed visually during the measurements: Spectra that obviously do not meet quality requirements are not stored.

In cases where a sufficient quality of spectra could not be achieved immediately, the RKI spectrum acquisition guidelines require a new sample preparation which includes variation of the analyte-matrix ratio. The best spectra were then selected from these measurement series. Important parameters for the visual assessment of spectrum quality were (i) the number of peaks contained in the respective spectrum, (ii) the signal-to-noise ratio, (iii) an ideally flat baseline, and (iv) a high resolving power of mass peaks.

As challenges for accurate identification include not only incomplete databases and close relatedness of the bacterial species of interest, but also insufficient spectral quality^43–45^, it was important for us to maintain high spectral quality over very long periods of time. Therefore, quality-relevant parameters of the spectrometer were checked and adjusted if necessary at longer intervals and after maintenance and repair of the instrument (e.g. laser replacement). Furthermore, an additionally installed focus lens for the beam path of the N_2_ laser installed at the RKI’s *autofleX I* instrument allowed precise adjustment of the laser focus position. The optimization of the position of this lens had a major influence on the spectrum quality. Other important instrument parameters with a significant influence on the spectrum quality were, among others, the condition of the ion source (regular cleaning required), the pulsed ion extraction time, and the amplifier voltage settings of the multichannel plate detector.

### Spectral quality testing

Starting with version 4 of the ZENODO database (20230306), all spectra stored in the database were additionally subjected to an automated software-based quality test. For this purpose, a specially developed software routine of the MicrobeMS software was employed. A total of four criteria were checked by these quality tests (QT): (i) number of peaks per spectrum, (ii) signal-to-noise ratio, (iii) baseline shape and (iv) mean resolving power of the identified mass peaks. During the quality tests, each spectrum is thus subjected to four independent tests, with individual scores determined for each QT criterion (0 - poor quality, test failed, 100 - excellent quality). A weighted overall QT score was then calculated from the scores. QT parameters are adjustable through the software, but were kept frozen after an initial testing phase and have not been changed since.

**Fig. 4:**
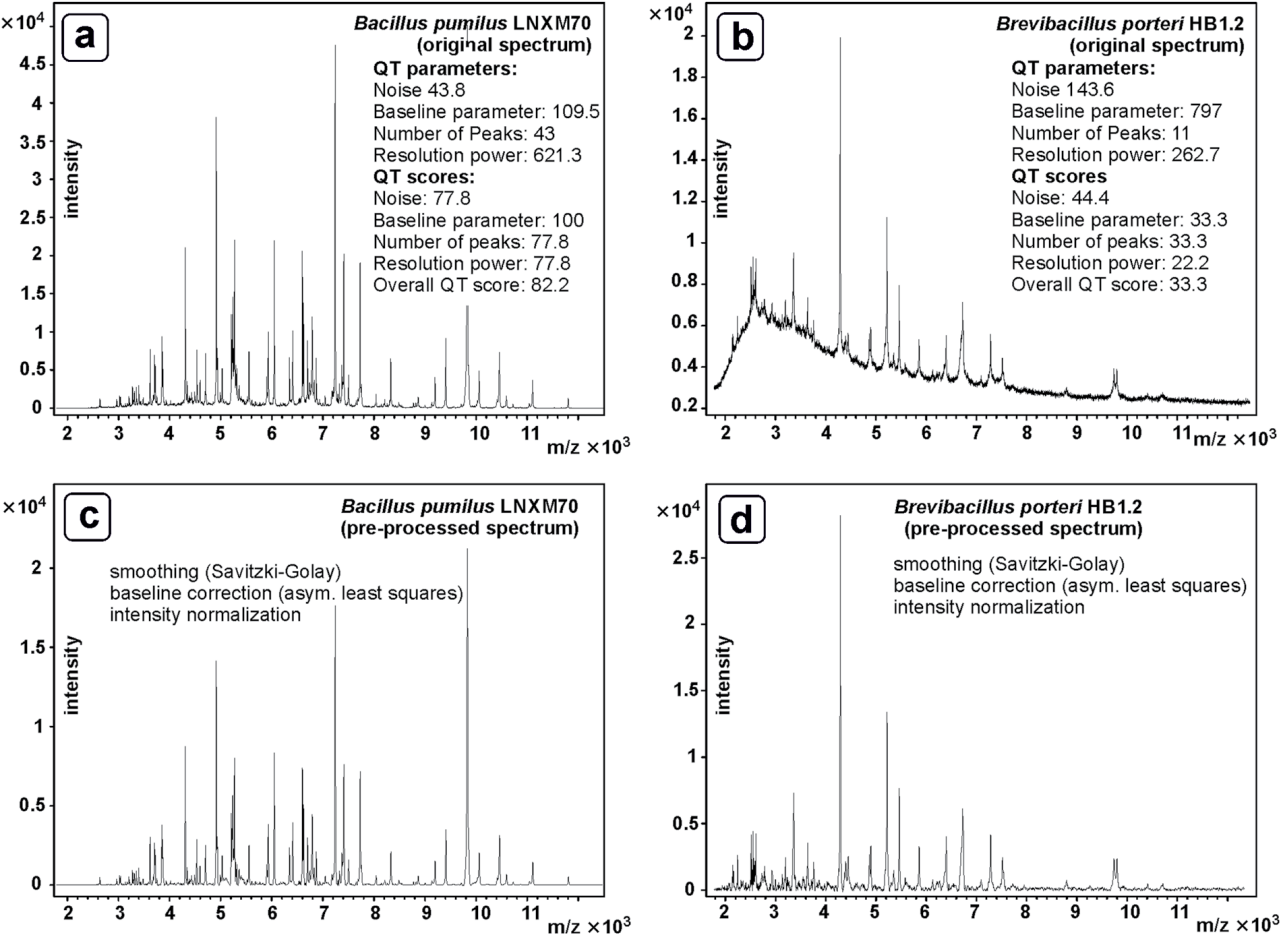
Comparison of high and moderate quality MALDI-ToF mass spectra. (**a**) High quality original spectrum obtained from strain *Bacillus pumilus* LNXM70. This spectrum shows relatively little noise, a flat baseline curve and many peaks with high resolving power. The overall quality test (QT) score reached a value of 82.2 (cf. inset for more details). The QT score may vary between values of 0 (poor quality) and 100 (excellent quality) (**b**) unprocessed MALDI-ToF mass spectrum acquired from a *Brevibacillus porteri* HB1.2 preparation showing an enhanced noise level, an elevated baseline in the low m/z region and less peaks of lower resolution. In this example, a QT score of 33.3 was determined. (**c**) processed spectrum derived from the original spectrum of panel a by smoothing, baseline correction and intensity normalization. (**d**) Preprocessed spectrum of panel b. As shown by this example, data preprocessing improves the apparent spectrum quality and is therefore important for a more accurate peak detection. This ultimately increases the taxonomic resolution and, as a result, the overall accuracy of the microbiological diagnostic method.

Figure 4 shows examples of two original and pre-treated MALDI-ToF mass spectra with high (*Bacillus pumilus* LNXM70, left column, panels a and c) and relatively low (*Brevibacillus porteri* HB 1.2, right column, panels b and d) spectral quality. For assessing the absolute noise level, original mass spectra were first automatically baseline corrected by means of the asymmetric least squares method^46^ and then vector normalized based on peak intensities. Noise was automatically determined from the spectra as the standard deviation of intensity values taken from m/z regions devoid of peaks. To determine the number of peaks of a given spectrum as a QT criterion, a minimum intensity function that candidate peaks must reach is determined. This function has a sigmoidal shape and is defined, among other factors, by the determined noise level. It is noteworthy that the number of peaks for QT and the number of peaks used for identification analysis can be very different because the latter routine uses pre-processed spectra that are of much higher quality compared to raw unprocessed spectra (less noise, flat baseline, see Fig. 4b,d).

The insets of Fig. 4a,b show the absolute QT values determined by MicrobeMS and the QT scores derived from them. When analyzing this figure, it is immediately apparent that the overall quality of the *Bacillus pumilus* LNXM70 spectrum (left, panel a) is significantly higher compared to the MALDI spectrum of *Brevibacillus porteri* HB 1.2 (right, panel b). However, despite the comparatively low QT score of 33.3, the spectrum of *Brevibacillus porteri* was still considered useful for database construction. We have observed that such spectra still allow extracting peak lists suitable for compiling useful MSPs and for accurate identification analysis.

**Fig. 5:**
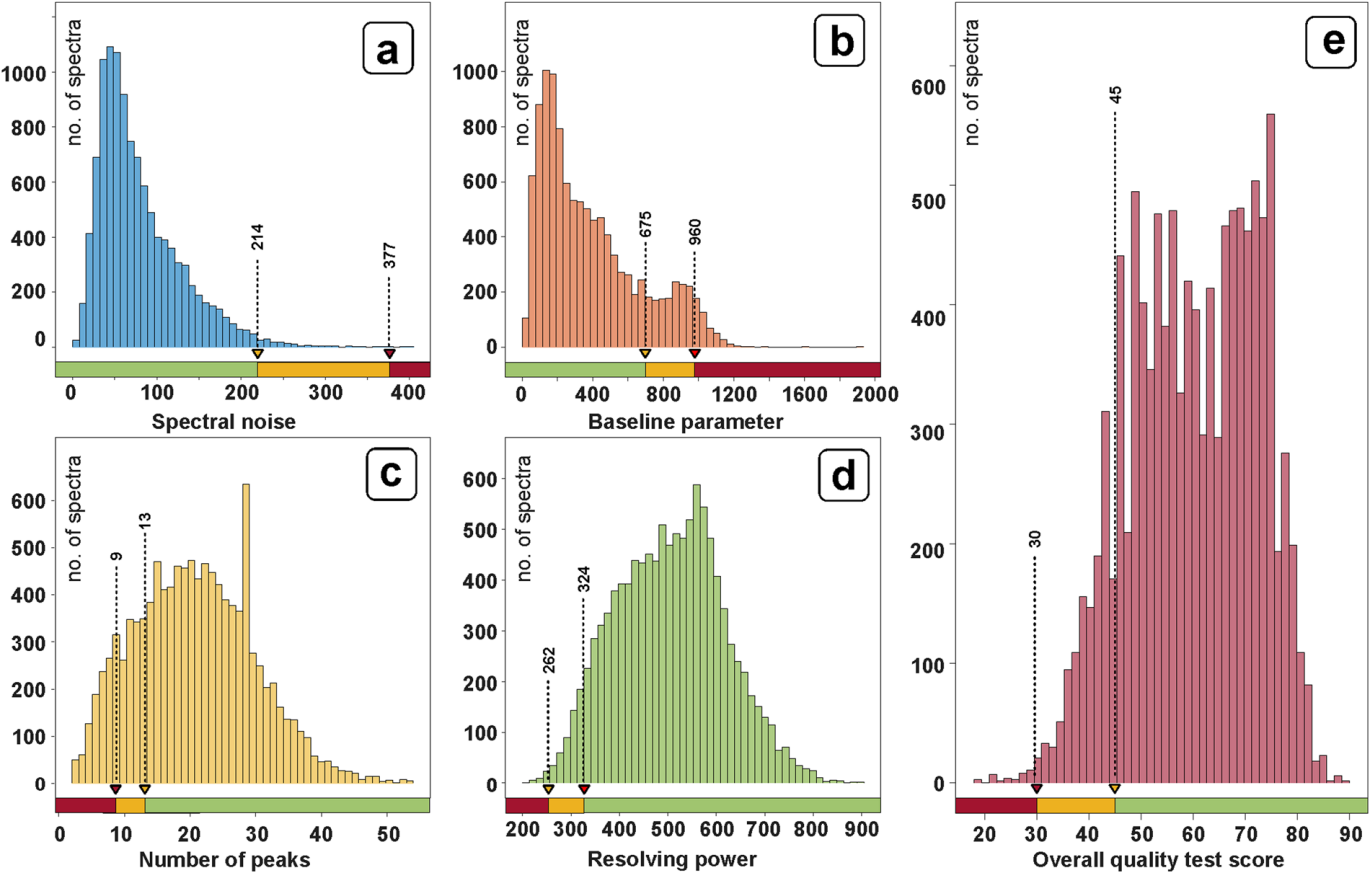
Results of quality tests (QT) of microbial MALDI-ToF mass spectra. Altogether, 11,055 mass spectra of the ZENODO v.4.2 MALDI-ToF MS database^41^ were tested and the respective QT parameters were obtained and analyzed. (**a**) Histogram showing the distribution of noise quality tests. Noise parameter was calculated by first ranking all data points of a spectrum by their intensity values in descending order. Subsequently, noise was calculated as the standard deviation from the lower 60% of all data points. Baseline correction and intensity normalization was carried out beforehand. (**b**) Histogram showing the frequency distribution of the baseline criterion. (c) Distribution of the QT criterion number of peaks (see text for details). (**d**) Parameter distribution of the resolving power tests. (**e**) Histogram of the overall QT results which are derived from the four QT parameters (i) noise, (ii) baseline parameter, (III) the number of peaks, and (iv) resolving power. For more information on QT scores computations, please refer to the MicrobeMS wiki page^43^.

The histograms of Fig. 5 provide a summary of the spectral quality of all MALDI-ToF mass spectra composing the database. While panels a-d depict the frequency distributions of the scores derived from the individual quality tests, panel e shows a histogram with the distribution of the total QT scores. It can be seen that more than 85% of all spectra in the database exhibit a total QT score higher than 45 (green in the traffic light scheme) and only less than 1.5% show a QT score below 30 (red). For planned future versions of the ZENODO database, we are currently in the process of gradually replacing spectra with low QT scores by spectra of higher quality.

### Spectral reproducibility

Bacterial preparations for MALDI-ToF MS identification analysis are inhomogeneous mixtures, resulting in a certain degree of spectral variance. In order to counteract only moderate spectral reproducibility, some authors recommend recording a larger number of spectra per individual sample when building a database. For example, to construct an MSP, Kostrzewa and Maier propose the acquisition of 24 technical replicate spectra, of which at least 20 should pass the QT^8^. In compiling the ZENODO database, we generally adhered to this recommendation, but also chose to deviate from some of the suggested points. While Kostrzewa and Maier only mention technical replicates, we generally aim to generate database spectra from three microbial cultures, from which 12 spectra ideally should be obtained (i.e. three biological replicates and 3 × 4 technical replicate spectra per strain). However, this value is not consistently reached due to limited personnel and time resources: the current database version contains an average of seven individual spectra per database entry. Although this number is below the recommended target value, it still allows for a high level of identification accuracy. For future database versions we will not only investigate new strains, species and genera, but also collect more spectra from already characterized isolates, especially from strains for which only spectra from one biological replicate are available so far.

The generation of representative database spectra, i.e. MSPs, is clearly influenced not only by the number of individual spectra per database entry. Other factors, such as the coverage of biological and technical variability^45^, high spectral quality^44^, and standardized procedures for sample preparation and spectra acquisition, to mention some factors, also have a significant influence on the accuracy and robustness of identification. In the current database version, the majority of samples were processed using the TFA inactivation and extraction method. In addition to the TFA method, the ethanol-formic acid (eth-FA) protocol recommended by Bruker and widely used in the MBT solution was employed as a supplementary sample preparation method. The comparability of spectra from both acid-based extraction protocols is of vital importance for potential users of our databases. Over the course of numerous years of investigation and systematic testing, we have discovered that spectra from both protocols exhibit remarkable similarities. Consequently, spectral databases obtained by the TFA method can be utilized to identify samples processed with the eth-FA protocol (and vice versa, see also discussion in Lasch *et al*.^15^).

### Calibration accuracy

Strictly speaking, the accuracy of calibration of the MALDI-ToF mass spectra can be viewed as an additional criterion for assessing the spectra quality. Unfortunately, the external calibration method employed – we used Bruker’s Protein Calibration Standard I, or a preparation of *Escherichia coli* DSM 3871 as external calibrants - does not allow for a universal calibration test. For such a test, all the microbial mass spectra examined would have to contain at least two universal mass peaks. As such universal mass peaks are not available, a subset of mass spectra from the Bacillus cereus group was selected to assess the accuracy of the calibration of the ZENODO database. This subset includes highly relevant species from the *Bacillus cereus* group, *Bacillus cereus* sensu stricto, *Bacillus thuringiensis*, and *Bacillus anthracis*, among some others. MALDI spectra from *B. cereus* group members exhibit at least three group-specific peaks^16^ and were acquired in large numbers (2442 in total, ~ 22% of all database spectra) by different operators over a period of almost 20 years. The calibration accuracy derived from the analysis of this subset selection can therefore be considered as being representative of the entire database.

**Fig. 6:**
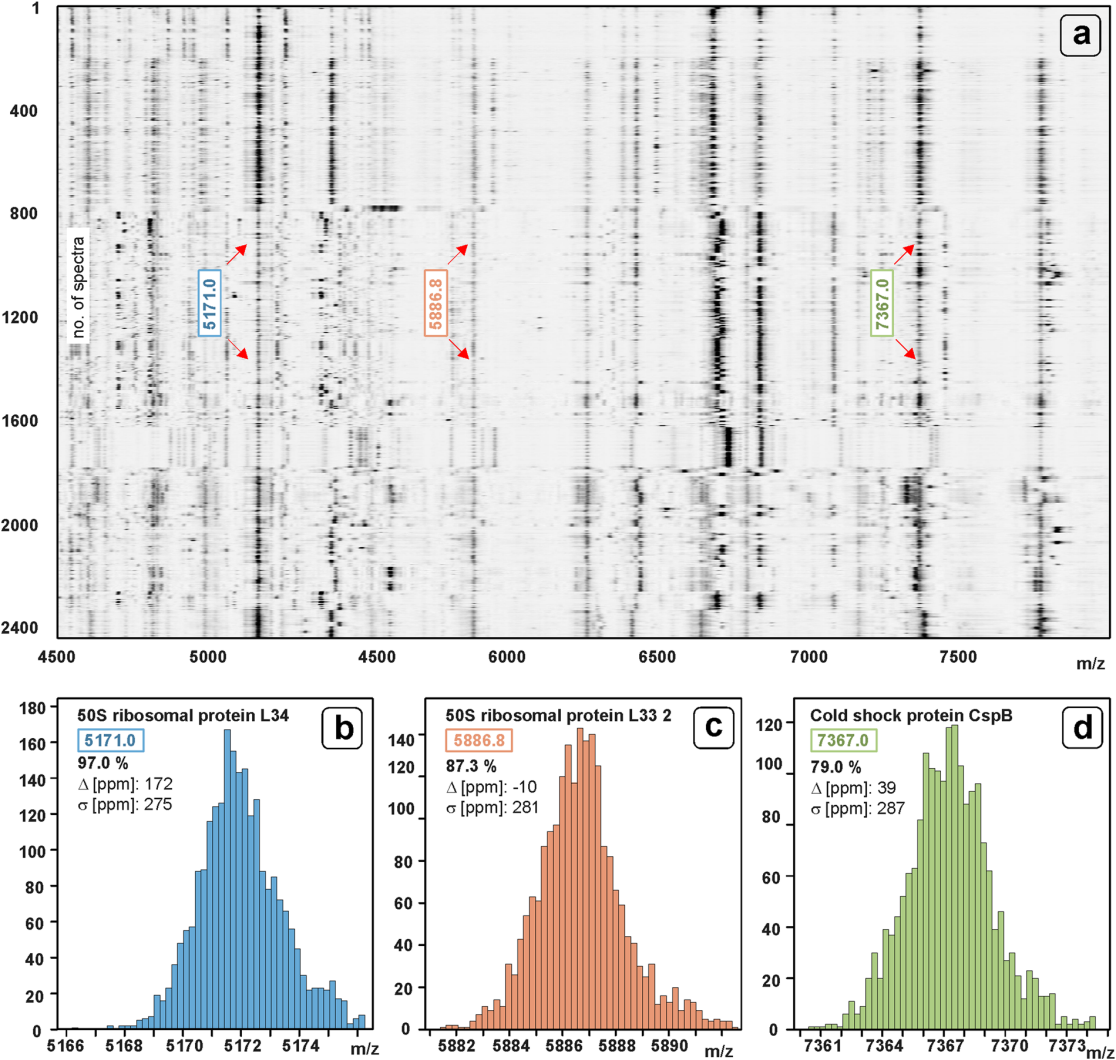
Quality of external calibration, exemplary illustrated by data from 2442 MALDI-ToF mass spectra of *Bacillus cereus* group strains. (**a**) Pseudo gel view between m/z 4500–8000 obtained from pre-processed (smoothed, baseline subtracted and intensity normalized) mass spectra. *B. cereus* group-specific MALDI signals are seen at m/z positions 5171 (50S ribosomal protein L34, blue), 5887 (50S ribosomal protein L33 2, peach) and 7367 (Cold shock protein CspB, avocado). (**b**) Histogram illustrating the distribution of precisely determined experimental positions of the peak assigned to L34, theoretical mass [M + H]^+^_theo_: 5171.10. The histogram inset shows the percentage of *B. cereus* group spectra of which the peak could be determined, the difference Δ (in ppm units) between the theoretical m/z position and the mean experimental m/z value and the standard deviation δ of the experimental peak positions (also in ppm units). (**c**) Histogram demonstrating the distribution of the peak positions around 5887 (L33 2, [M + H]^+^_theo_: 5886.79). (**d**) Distribution of peak positions at 7367, [M + H]^+^_theo_: 7367.03.

The quality of the external calibration of *B. cereus* group database spectra is illustrated by Fig. 6. Figure 6a shows a pseudo-gel view of pre-processed mass spectra in the m/z range of 4500–8000. It can be seen that MALDI-ToF mass spectra of *B. cereus* group members exhibit signals at m/z positions 5171 (50S ribosomal protein L34), 5887 (50S ribosomal protein L33 2, peach) and 7367 (cold shock protein CspB, see arrows in panel a)^16,47^. The histograms in Fig. 6b–d illustrate the distribution of m/z positions of the respective mass peaks assigned to 50S ribosomal protein L34 (panel b), 50S ribosomal protein L33 2 (c), and cold shock protein CspB (d). The inset text indicates the mean peak position derived from the experimental mass spectra, the percentage frequency of the given peak, the difference Δ between the theoretical and experimental m/z values, and the standard deviation δ of the experimental peak positions. The average experimental peak positions deviate from the theoretical values by less than 200 ppm and show δ-values below 300 ppm, which is well below the usually reported threshold of 800 ppm.

### Taxonomic accuracy

Correct taxonomical assignment is paramount when compiling spectral databases. The species, or subspecies identity of the bacterial strains of the database was thus determined with great care using different, well-established bacterial characterization techniques such as phenotyping methods, 16S rRNA sequencing, WGS, and in selected cases by proteomics (liquid chromatography - mass spectrometry, LC-MS) methods used^48,49^ or developed^50^ by authors of this study. Furthermore, a large fraction of the bacteria studied originate from public strain collections such as the German Collection of Microorganisms and Cell Cultures (DSMZ), American Type Culture Collection (ATCC), Laboratorium voor Microbiologie Ghent (LMG), National Type Culture Collection (NTCC), to name but a few. In recent years, strains from the microorganism collection of the RKI have been completely taxonomically characterized by WGS. The same applies to the spectra of HBP and their relatives provided by most of our partner institutions.

In addition, in order to avoid erroneous database entries, all spectra available in the database have been checked for identity using the latest version of Bruker’s MBT solution. Questionable identification results were systematically investigated and, in the case of discrepancies, the corresponding entries were removed from the database. Moreover, feedback from users of previous database versions was thoroughly reviewed and any database spectrum with questionable species identity was removed.

## Usage Notes

The published MALDI-ToF mass spectra are expected to be of interest to the broader clinical microbiology community working with BSL-3 pathogens. We expect that our growing ZENODO dataset will provide a reference for experimentally measured MALDI-ToF mass spectra of HPB and thus contribute to improved characterization and identification of these pathogens in clinical diagnostics and research. The spectrum collection will also be valuable for other fields of work, such as veterinary medicine or food control. Furthermore, the publication of the dataset aims to provide the bio- and cheminformatics community with a large number of annotated high-quality and well-characterized mass spectra. These could be used, for example, to develop and test new or improved algorithms for identification analysis or further characterization efforts such as antibiotic resistance, or virulence factor detection. The database solutions MBT, VITEK – MS, and Andromas offered by vendors such as Bruker, bioMérieux, or Andromas are known to be closed commercial systems where the scientific community does not have access to the underlying source data. As a result, the high quality and extensive data collected by the commercial vendors is not available to the scientific community, which means that this data cannot be used for developing and evaluating new identification approaches. We hope that our publication will facilitate such development work and, therefore, intend to further expand and advance the ZENODO database in the future.

Original MALDI-ToF mass spectra in the manufacturer-specific data format (Bruker Daltonics) are available with no restrictions from ZENODO (zenodo db 230306.zip) as a zip archive and can be handled by Bruker software such as FlexAnalysis, ClinProTools and the MBT software in their current versions. Data are published under a Creative Commons Attribution Non-Commercial 4.0 International (CC-BY-NC): Licensees must credit the original authors by stating their names & the original work’s title. Licensees may copy, distribute, display, and perform the work and make derivative works and remixes based on it for non-commercial purposes.

Although the spectra have been recorded with the greatest care and tested extensively, the database entries should be checked before use. It is strongly recommended that any diagnostic results obtained from the use of this database are verified by an alternative method. No liability can be accepted under any circumstances.

The taxonomic names of the bacterial isolates examined reflect the status in the scientific literature as of May 2023.

## Data Availability

No custom code was used during this study for the curation and/or validation of the MALDI-ToF MS dataset. The Matlab-based software MicrobeMS^43^ used for this work is freely available as Matlab pcode from https://wiki-ms.microbe-ms.com.

## References

[1] Cuenod, A. *et al*. Quality of MALDI-TOF mass spectra in routine diagnostics: results from an international external quality assessment including 36 laboratories from 12 countries using 47 challenging bacterial strains. *Clin. Microbiol. Infect*. 10.1016/j.cmi.2022.05.017 (2022). 10.1016/j.cmi.2022.05.017 35623578

[2] Welker, M., Van Belkum, A., Girard, V., Charrier, J. P. & Pincus, D. An update on the routine application of MALDI-TOF MS in clinical microbiology. *Expert Rev Proteomics* **16**, 695–710, 10.1080/14789450.2019.1645603 (2019). 31315000 10.1080/14789450.2019.1645603

[3] Sauer, S. & Kliem, M. Mass spectrometry tools for the classification and identification of bacteria. *Nat. Rev. Microbiol.* **8**, 74–82, 10.1038/nrmicro2243 (2010). 20010952 10.1038/nrmicro2243

[4] Maier, T., Klepel, S., Renner, Z. & Kostrzewa, M. Fast and reliable MALDI-TOF MS-based microorganism identification. *Nat. Methods.* **3**, 324–334, 10.1038/nmeth870 (2006).

[5] Seng, P. *et al*. Ongoing revolution in bacteriology: routine identification of bacteria by matrix-assisted laser desorption ionization time-of-flight mass spectrometry. *Clin. Infect. Dis.* **49**, 543–551, 10.1086/600885 (2009). 19583519 10.1086/600885

[6] Clark, A. E., Kaleta, E. J., Arora, A. & Wolk, D. M. Matrix-assisted laser desorption ionization-time of flight mass spectrometry: a fundamental shift in the routine practice of clinical microbiology. *Clin. Microbiol. Rev.* **26**, 547–603, 10.1128/CMR.00072-12 (2013). 23824373 10.1128/CMR.00072-12 PMC3719498

[7] Mortier, T., Wieme, A. D., Vandamme, P. & Waegeman, W. Bacterial species identification using MALDI-TOF mass spectrometry and machine learning techniques: A large-scale benchmarking study. *Comput Struct Biotechnol J* **19**, 6157–6168, 10.1016/j.csbj.2021.11.004 (2021). 34938408 10.1016/j.csbj.2021.11.004 PMC8649224

[8] Kostrzewa, M. & Maier, T. Criteria for Development of MALDI-TOF Mass Spectral Database. (2017).

[9] Ashfaq, M. Y., Da’na, D. A. & Al-Ghouti, M. A. Application of MALDI-TOF MS for identification of environmental bacteria: A review. *J. Environ. Manage.* **305**, 114359, 10.1016/j.jenvman.2021.114359 (2022). 34959061 10.1016/j.jenvman.2021.114359

[10] de Koster, C. G. & Brul, S. MALDI-TOF MS identification and tracking of food spoilers and food-borne pathogens. *Current Opinion in Food Science* **10**, 76–84, 10.1016/j.cofs.2016.11.004 (2016).

[11] Thompson, J. E. Matrix-assisted laser desorption ionization-time-of-flight mass spectrometry in veterinary medicine: Recent advances (2019-present). *Vet World* **15**, 2623–2657, 10.14202/vetworld.2022.2623-2657 (2022). 36590115 10.14202/vetworld.2022.2623-2657 PMC9798047

[12] Elhanany, E., Barak, R., Fisher, M., Kobiler, D. & Altboum, Z. Detection of specific Bacillus anthracis spore biomarkers by matrix-assisted laser desorption/ionization time-of-flight mass spectrometry. *Rapid Commun. Mass Spectrom.* **15**, 2110–2116, 10.1002/rcm.491 (2001). 11746875 10.1002/rcm.491

[13] Lasch, P. *et al*. Identification of Highly Pathogenic Microorganisms by Matrix-Assisted Laser Desorption Ionization-Time of Flight Mass Spectrometry: Results of an Interlaboratory Ring Trial. *J. Clin. Microbiol.* **53**, 2632–2640, 10.1128/JCM.00813-15 (2015). 26063856 10.1128/JCM.00813-15 PMC4508426

[14] Lasch, P. *et al*. MALDI-TOF mass spectrometry compatible inactivation method for highly pathogenic microbial cells and spores. *Anal. Chem.* **80**, 2026–2034, 10.1021/ac701822j (2008). 18290666 10.1021/ac701822j

[15] Lasch, P., Grunow, R., Antonation, K., Weller, S. A. & Jacob, D. Inactivation Techniques for MALDI-TOF MS Analysis of Highly Pathogenic Bacteria - A Critical Review. *Trac-Trend Anal Chem* **85**, **Part B**, 112–119 (2016). 10.1016/j.trac.2016.04.012.

[16] Lasch, P. *et al*. Identification of Bacillus anthracis by using matrix-assisted laser desorption ionization-time of flight mass spectrometry and artificial neural networks. *Appl. Environ. Microbiol.* **75**, 7229–7242, 10.1128/AEM.00857-09 (2009). 19767470 10.1128/AEM.00857-09 PMC2786504

[17] Lasch, P. *et al*. Characterization of Yersinia using MALDI-TOF mass spectrometry and chemometrics. *Anal. Chem.* **82**, 8464–8475, 10.1021/ac101036s (2010). 20866090 10.1021/ac101036s

[18] Lasch, P. & Naumann, D. MALDI-TOF Mass Spectrometry for the Rapid Identification of Highly Pathogenic Microorganisms. *Proteomics, Glycomics and Antigenicity of BSL3 and BSL4 Agents, First Edition. Edited by Jiri Stulik, Rudolf Toman, Patrick Butaye, Robert G. Ulrich. 2011 Wiley-VCH Verlag GmbH & Co. KGaA. Published 2011 by Wiley-VCH Verlag GmbH & Co. KGaA*., 219-212 (2011).

[19] Minan, A. *et al*. Rapid identification of Burkholderia cepacia complex species including strains of the novel Taxon K, recovered from cystic fibrosis patients by intact cell MALDI-ToF mass spectrometry. *Analyst.* **134**, 1138–1148, 10.1039/b822669e (2009). 19475140 10.1039/b822669e

[20] Martina, P. *et al*. Burkholderia puraquae sp. nov., a novel species of the Burkholderia cepacia complex isolated from hospital settings and agricultural soils. *Int. J. Syst. Evol. Microbiol.* **68**, 14–20, 10.1099/ijsem.0.002293 (2018). 29095137 10.1099/ijsem.0.002293

[21] Wu, H. *et al*. Cold-adapted Bacilli isolated from the Qinghai-Tibetan Plateau are able to promote plant growth in extreme environments. *Environ. Microbiol*. **0**10.1111/1462-2920.14722 (2019). 10.1111/1462-2920.14722 31233661

[22] Tam, L. T. T. *et al*. Draft Genome Sequences of 59 Endospore-Forming Gram-Positive Bacteria Associated with Crop Plants Grown in Vietnam. *Microbiol Resour Announc* **9**10.1128/MRA.01154-20 (2020). 10.1128/MRA.01154-20 PMC7679102 33214309

[23] Karger, A. *et al*. Rapid identification of Burkholderia mallei and Burkholderia pseudomallei by intact cell Matrix-assisted Laser Desorption/Ionisation mass spectrometric typing. *BMC Microbiol.* **12**, 229, 10.1186/1471-2180-12-229 (2012). 23046611 10.1186/1471-2180-12-229 PMC3534143

[24] Lista, F. *et al*. Reliable identification at the species level of Brucella isolates with MALDI-TOF-MS. *BMC Microbiol.* **11**, 267, 10.1186/1471-2180-11-267 (2011). 22192890 10.1186/1471-2180-11-267 PMC3314589

[25] Contzen, M., Hailer, M. & Rau, J. Isolation of Bacillus cytotoxicus from various commercial potato products. *Int. J. Food Microbiol.* **174**, 19–22, 10.1016/j.ijfoodmicro.2013.12.024 (2014). 24440535 10.1016/j.ijfoodmicro.2013.12.024

[26] Abdelli, M. *et al*. Get to Know Your Neighbors: Characterization of Close Bacillus anthracis Isolates and Toxin Profile Diversity in the Bacillus cereus Group. *Microorganisms* **11**, 2721, 10.3390/microorganisms11112721 (2023). 38004733 10.3390/microorganisms11112721 PMC10673079

[27] Mostaghat, I. *et al*. Management of unexpected laboratory exposure to Burkholderia pseudomallei. *Ann. Biol. Clin. (Paris)* **81**, 640–644, 10.1684/abc.2023.1854 (2024). 38391168 10.1684/abc.2023.1854

[28] Nozaki, Y. *et al*. A case of renal abscess and bacteremia caused by Burkholderia pseudomallei that was first unidentifiable by matrix-assisted laser desorption ionization-time of flight mass spectrometry in a Japanese-man. *J Infect Chemother*10.1016/j.jiac.2021.06.005 (2021). 34147356 10.1016/j.jiac.2021.06.005

[29] Walewski, V. *et al*. MALDI-TOF MS contribution to diagnosis of melioidosis in a nonendemic country in three French travellers. *New Microbes New Infect* **12**, 31–34, 10.1016/j.nmni.2016.04.004 (2016). 27222715 10.1016/j.nmni.2016.04.004 PMC4872369

[30] Howley, F., Abukhodair, S., de Barra, E., O’Connell, K. & McNally, C. Misidentification of Brucella melitensis as Ochrobactrum species: potential pitfalls in the diagnosis of brucellosis. *BMJ Case Rep*. **17**10.1136/bcr-2024-260072 (2024). 10.1136/bcr-2024-260072 38901850

[31] Doellinger, J., Schneider, A., Stark, T., Ehling-Schulz, M. & Lasch, P. Evaluation of MALDI-ToF Mass Spectrometry for Rapid Detection of Cereulide from Bacillus cereus Cultures. *bioRxiv*. 10.1101/869958 (2019). 10.3389/fmicb.2020.511674 PMC7709880 33329410

[32] Lasch, P. *et al*. Insufficient discriminatory power of MALDI-TOF mass spectrometry for typing of Enterococcus faecium and Staphylococcus aureus isolates. *J. Microbiol. Methods* **100**, 58–69, 10.1016/j.mimet.2014.02.015 (2014). 24614010 10.1016/j.mimet.2014.02.015

[33] Lasch, P., Jacob, D., Klee, S. R. & Werner, G. Discriminatory Power of MALDI-TOF Mass Spectrometry for Phylogenetically Closely Related Microbial Strains. In: *Applications of Mass Spectrometry in Microbiology, Plamen Demirev*, Todd R. Sandrin (Eds.) Springer International Publishing, 203–234 (2016).

[34] Dieckmann, R. *et al*. Rapid characterisation of Klebsiella oxytoca isolates from contaminated liquid hand soap using mass spectrometry, FTIR and Raman spectroscopy. *Faraday Discuss.* **187**, 353–375, 10.1039/c5fd00165j (2016). 27053001 10.1039/c5fd00165j

[35] Rau, J. *et al*. MALDI-UP–An internet platform for the exchange of MALDI-TOF mass spectra. *Asp Food Contr Anim Health* **01** (2016).

[36] Park, J. H., Kim, T. S., Park, H. & Kang, C. K. Delay in the diagnosis of Brucella abortus bacteremia in a nonendemic country: a case report. *BMC Infect. Dis.* **24**, 489, 10.1186/s12879-024-09377-y (2024). 38741035 10.1186/s12879-024-09377-y PMC11089730

[37] Suniga, P. A. P. *et al*. Glanders Diagnosis in an Asymptomatic Mare from Brazil: Insights from Serology, Microbiological Culture, Mass Spectrometry, and Genome Sequencing. *Pathogens* **12**10.3390/pathogens12101250 (2023). 10.3390/pathogens12101250 PMC10609850 37887766

[38] Levasseur, M. *et al*. Classification of Environmental Strains from Order to Genus Levels Using Lipid and Protein MALDI-ToF Fingerprintings and Chemotaxonomic Network Analysis. *Microorganisms* **10**10.3390/microorganisms10040831 (2022). 10.3390/microorganisms10040831 PMC9032901 35456880

[39] Alexandre, G. *et al*. MSclassifR: an R Package for Supervised Classification of Mass Spectra with Machine Learning Methods. *bioRxiv*, 2022.2003.2014.484252 10.1101/2022.03.14.484252 (2023).

[40] De Waele, G., Menschaert, G., Vandamme, P. & Waegeman, W. Pre-trained Maldi Transformers improve MALDI-TOF MS-based prediction. *bioRxiv*, 2024.2001.2018.576189 10.1101/2024.01.18.576189 (2024). 10.1016/j.compbiomed.2025.109695 39847945

[41] Lasch, P., Stämmler, M. & Schneider, A. Version 4.2 (20230306) of the MALDI-ToF Mass Spectrometry Database for Identification and Classification of Highly Pathogenic Microorganisms from the Robert Koch-Institute (RKI). *Zenodo* **March 6, 2023**10.5281/zenodo.14562231 (2023).

[42] Freiwald, A. & Sauer, S. Phylogenetic classification and identification of bacteria by mass spectrometry. *Nat. Protoc.* **4**, 732–742, 10.1038/nprot.2009.37 (2009). 19390529 10.1038/nprot.2009.37

[43] Lasch, P. MicrobeMS - A Matlab Toolbox for Microbial Identification Based on Mass Spectrometry. https://wiki-ms.microbe-ms.com, **last accessed Dec 31, 2024** (2024).

[44] Cuenod, A., Foucault, F., Pfluger, V. & Egli, A. Factors Associated With MALDI-TOF Mass Spectral Quality of Species Identification in Clinical Routine Diagnostics. *Front Cell Infect Microbiol* **11**, 646648, 10.3389/fcimb.2021.646648 (2021). 33796488 10.3389/fcimb.2021.646648 PMC8007975

[45] Oberle, M. *et al*. The Technical and Biological Reproducibility of Matrix-Assisted Laser Desorption Ionization-Time of Flight Mass Spectrometry (MALDI-TOF MS) Based Typing: Employment of Bioinformatics in a Multicenter Study. *PLoS One* **11**, e0164260, 10.1371/journal.pone.0164260 (2016). 27798637 10.1371/journal.pone.0164260 PMC5087883

[46] Eilers, P. H. C. & Boelens, H. F. M. Baseline Correction with Asymmetric Least Squares Smoothing. *Leiden University Centre Medical Report* **1**, 5 (2005).

[47] Lasch, P., Jacob, D., Grunow, R., Schwecke, T. & Doellinger, J. Matrix-assisted laser desorption/ionization time-of-flight (MALDI-TOF MS for the identification of highly pathogenic bacteria. *Trac-Trend Anal Chem* **85**, Part B, 103–111 (2016).

[48] Mesuere, B. *et al*. Unipept: tryptic peptide-based biodiversity analysis of metaproteome samples. *J. Proteome Res.* **11**, 5773–5780, 10.1021/pr300576s (2012). 23153116 10.1021/pr300576s

[49] Verschaffelt, P. *et al*. Unipept Visualizations: an interactive visualization library for biological data. *Bioinformatics* **38**, 562–563, 10.1093/bioinformatics/btab590 (2022). 34390575 10.1093/bioinformatics/btab590

[50] Lasch, P., Schneider, A., Blumenscheit, C. & Doellinger, J. Identification of Microorganisms by Liquid Chromatography-Mass Spectrometry (LC-MS^1^) and in Silico Peptide Mass Libraries. *Mol. Cell. Proteomics* **19**, 2125–2139, 10.1074/mcp.TIR120.002061 (2020). 32998977 10.1074/mcp.TIR120.002061 PMC7710138

